# Impacts of the COVID‐19 pandemic on field instruction and remote teaching alternatives: Results from a survey of instructors

**DOI:** 10.1002/ece3.6628

**Published:** 2020-08-07

**Authors:** Daniel C. Barton

**Affiliations:** ^1^ Department of Wildlife Humboldt State University Arcata CA USA

**Keywords:** faculty survey, field instruction, pedagogy, remote teaching

## Abstract

Education in ecology and evolution often utilizes field instruction to teach key learning outcomes. Remote teaching of learning outcomes that have been traditionally taught in the field, necessitated by the COVID‐19 pandemic, presents unique challenges for students, instructors, and institutions. A survey of 117 faculty conducted during spring 2020 revealed substantial reduction of learning outcomes typically taught in the field, and frequent substitutions of less active and more instructor‐centered remote activities for field activities. The survey revealed generally negative instructor views on many remote teaching substitutions, yet also showed several approaches that instructors regarded as more effective, despite potential challenges with equitably teaching them. I suggest several models of remote substitutions for traditional field teaching of identification, field techniques, data collection, and study design in the context of the results of this survey.

## Graphical abstract

Remote teaching of learning outcomes that have been traditionally taught in the field, necessitated by the COVID‐19 pandemic, presents unique challenges for students, instructors, and institutions. A survey of 117 faculty conducted during spring 2020 revealed impacts to and potentially effective substitutions for field teaching appropriate for remote teaching modalities.

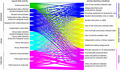

## 1 INTRODUCTION

Basic and applied disciplines rooted in ecology and evolution traditionally rely on experiential field instruction to teach key learning outcomes representing natural history, study design, field methods, and the process of scientific inquiry (Fleischner et al., 2017; Herman, 2002; Tewksbury et al., 2014). Other disciplines, such as the geosciences, similarly rely upon field activities in instruction (Whitmeyer & Mogk, 2009). Field activities, defined here as educational activities that occur outside and involve interaction with the natural or built environment (Fleischner et al., 2017), can provide unique and engaging instruction that is often vital to learning outcomes of postsecondary courses, even when they represent a relatively small portion of instruction (Harland, Spronken‐Smith, Dickinson, & Pickering, 2006; Hole, 2018). Potential impacts of reduction and elimination of field activities and natural history education from undergraduate curricula have been previously recognized (Tewksbury et al., 2014) as have potential solutions (Fleischner et al., 2017). Despite its potential importance, biology education research appears to have paid relatively little attention to postsecondary field teaching compared to classroom teaching (Singer, Nielsen, & Schweingruber, 2013) or relative to other disciplines (e.g. geography; Boyle et al., 2007).

The COVID‐19 pandemic (Fauci, Lane, & Redfield, 2020) has clearly posed a unique set of challenges to higher education (Sahu, 2020), and particularly to face‐to‐face field activities and the learning outcomes associated with them. Meeting these challenges may be hampered by a general lack of research on field pedagogy and the somewhat idiosyncratic nature of field teaching (Fleischner et al., 2017). The pandemic has highlighted an ongoing need for educational research on pedagogy in field settings (Singer et al., 2013), and immediately, for specific research focused on how instructors may be able to most effectively shift the teaching of important learning outcomes from face‐to‐face to remote teaching (or distance‐learning; hereafter, remote) modalities.

Rapid shifts from face‐to‐face modalities to remote modalities at US postsecondary institutions during spring 2020 clearly must have impacted field teaching activities on a large scale. I surveyed a sample of instructors of college‐level courses with field components during April and May 2020 to understand these impacts. The survey was designed to answer three inductive research questions: (a) What types of activities and learning outcomes were typically taught by instructors teaching in the field? (b) How did the shift in teaching modality immediately affect instruction of learning outcomes typically taught in field settings, and what types of activities did instructors use to substitute for field activities? (c) What are the major challenges and potential solutions to effectively and inclusively teaching learning outcomes typically taught in field settings in a remote modality? Here, I report the results of this survey and suggest several alternative approaches to remotely teaching field activities based on approaches being used by or planned by survey respondents.

## 2 METHODS

I collected email addresses of 2,000 faculty with field‐based specializations in applied and basic biological, environmental, and geophysical science using departmental websites of a nonrandom selection of 200 public and private research universities, undergraduate‐serving institutions, and community colleges located in the United States. The prospective respondents represented a nonrandom but wide range of institution types, disciplines, and geography. I then randomly selected an unstratified sample of 1,000 faculty for recruitment into the survey and contacted them via email between 16 April and 25 April 2020 (contact email details are in Supporting Information). A follow‐up email was sent 5 days later to nonrespondents. A unique link to the online survey was also emailed to several discipline‐ and interest‐specific mailing lists maintained by the Ecological Society of America, Pacific Seabird Group, and Society for the Advancement of Biology Education Research. The survey period closed May 10, 2020.

The survey was administered using the web platform SurveyMonkey (SurveyMonkey, 2020). The survey process included an informed consent statement, and consisted of 22 questions, not counting an informed consent acceptance and additional communication opt‐in (informed consent, complete survey questions, and response options are reported in Supporting Information). Respondents were notified that their individual responses were private, and individually identifiable survey data were anonymized and separated from survey responses before storage and analysis. Standard psychometric principles were not used in the creation of all survey questions given the backgrounds of the prospective respondent pool, and my intent to use these data in this purely descriptive or inductive study. Five of the 22 questions interrogated the respondent's current teaching and plans to teach courses, institutional and positional characteristics, and specific discipline. The remaining 17 questions interrogated the instructor's typical field instruction, and their perception of impacts of the COVID‐19 pandemic and teaching modality shifts on typical field instruction (Supporting Information; figures and tables reference specific questions by number). Respondents were informed that they could opt‐out at any time, and a final submission was required for their results to be recorded. One survey response was eliminated because it contained numerous extraneous and perhaps poorly intentioned responses. The remaining surveys were used to create simple descriptive statistics, and summary figures and tables. Not all respondents answered all questions, and thus, sample sizes varied among questions and are reported on a question‐by‐question basis. Aggregated and anonymized data are reported in Supporting Information. Individual responses to free‐response items are not shown as some contained potentially individually identifiable information, and informed consent was not obtained from subjects to use direct quotations of their responses. The only person with access to nonanonymized and nonaggregated data was DC Barton. The survey and data‐handling protocol were approved by the Humboldt State University Institutional Review Board for the Protection of Human Subjects (IRB #19‐164) in April 2020.

## 3 RESULTS

One hundred and seventeen respondents submitted a survey and represented at least 70 different institutions, including doctoral universities (51; 44.35%), master's colleges or universities (29; 25.22%), baccalaureate colleges (24; 20.87%), baccalaureate/associate's colleges (3; 2.61%), and associate's colleges (8; 6.96%) that were both public (92; 80%) and private (23; 20%). Respondents were largely tenure‐track or tenured faculty (93; 80.87%), but included lecturers or adjunct faculty (11; 9.57%), research faculty (4, 3.48%), graduate students (4; 3.48%), postdoctoral associates (1; 0.87%), administrators (1; 0.87%), and instructional support or faculty development staff (1; 0.87%).

Respondents reported primarily teaching courses in a variety of disciplines in field settings, categorized post hoc (see Supporting Information for complete list) as earth sciences (14; 13.1%), ecology, wildlife biology, and vertebrate zoology (52; 48.6%), fisheries, oceanography and marine biology (9; 8.4%), forestry, botany, and soil science (20; 18.7%), general biology (2; 1.9%), invertebrate zoology (2; 1.9%), outdoor education (4; 3.7%), and environmental science (4; 3.7%). Assessed learning outcomes of these courses were not dependent on field components (1; 0.9%), minimally dependent on field components (32; 27.6%), largely dependent on field components (74; 63.8%), or wholly dependent on field components (9; 7.76%). Students in these courses were composed of first‐year (21; 18.10%), second‐year (43; 37.1%), third‐year (60; 51.7%), fourth‐year and beyond (73; 62.9%), or graduate students (21; 18.1%). The types of field components in respondents’ courses included short field trips (90; 77.6%), day‐long field trips (42; 36.2%), overnight trips of less than three nights (27; 23.3%), overnight trips of three of more nights (15; 12.9%), courses largely or completely taught in the field or at a field station (24; 20.7%), and included supervised fieldwork conducted during field trips (48; 41.38%) as well as independent fieldwork conducted by students on their own time (45; 38.79%).

The majority of respondents (93; 79.5%) reported instructing courses with field components for which the mode of instruction was impacted by the COVID‐19 pandemic during spring 2020, and many respondents (53; 45.3%) anticipated impacts to courses with field components they plant to instruct in summer or fall 2020. Three respondents (2.56%) were already teaching remote courses in spring 2020 with field components before the onset of the pandemic, and five respondents (4.27%) were already planning to remotely teach a course with field components in summer 2020. Six respondents (5.13%) either instruct, have instructed, or develop instructional materials for courses with field components but were not currently teaching in spring, summer, or fall 2020. In response to the COVID‐19 pandemic, some (33; 29.0%) respondents reported removing or planning to remove and many (53; 46.5%) reported reducing or planning to reduce field learning outcomes. Most respondents (65; 57.0%) reported switching from teaching field learning outcomes in the field to teaching them remotely, and some (37; 32.5%) reported switching from teaching field learning outcomes in a typical field setting to teaching them remotely, but still in the field. One respondent (0.88%) reported making no changes because they were already teaching remotely.

**Figure 1:**
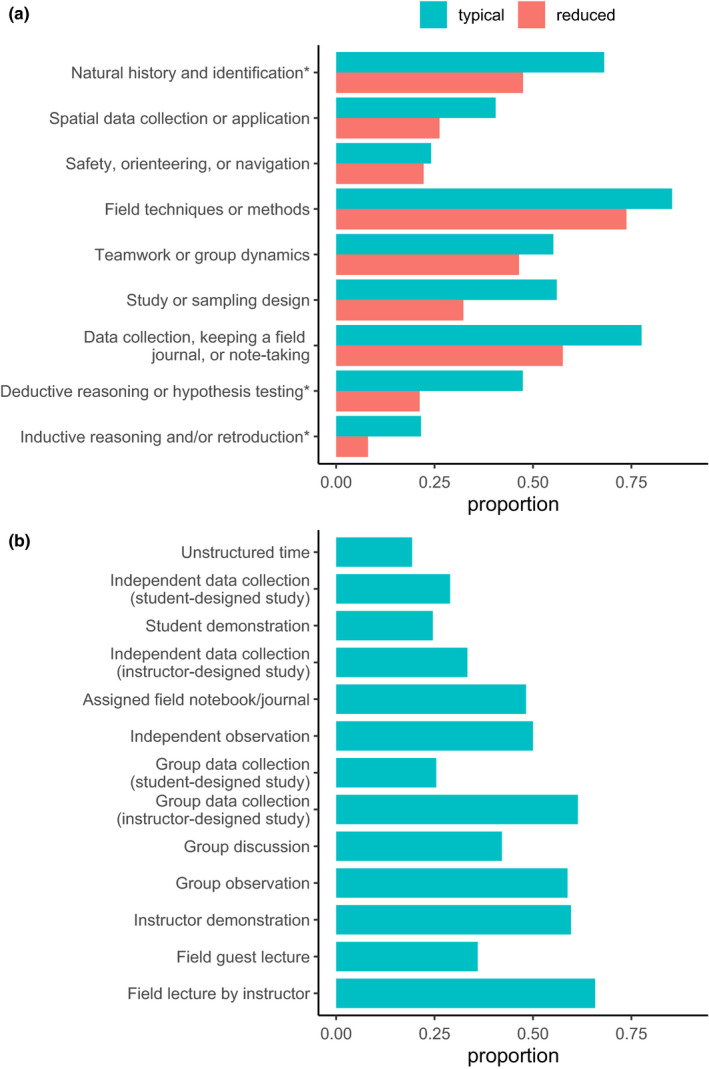
(a) Proportion of respondents that reported teaching a particular type of learning outcome and that reduced or eliminated particular learning outcomes because of the COVID‐19 pandemic (*n* = 116; questions #7 and #10). (b) Proportion of respondents that reported teaching a particular type of field learning activity prepandemic (*n* = 114; question #8)

**Figure 2:**
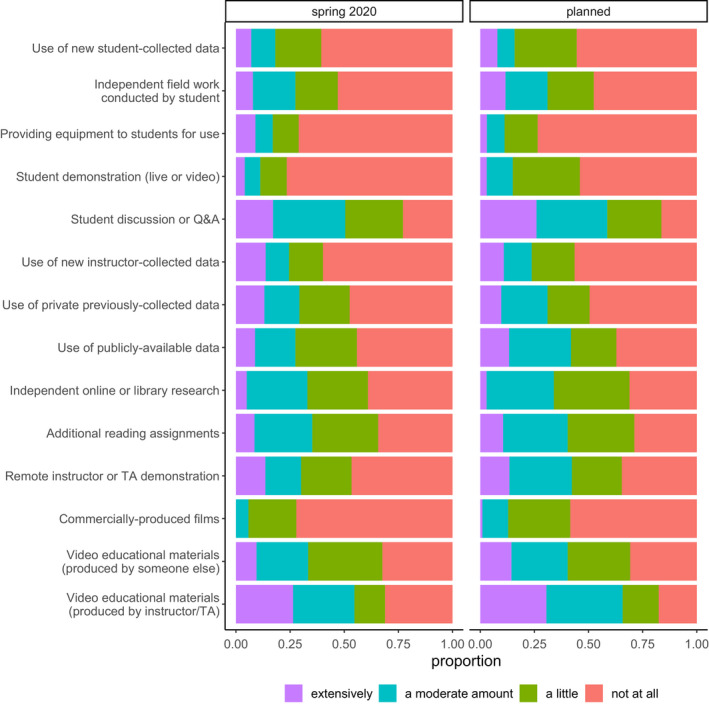
Extent of remote teaching activity usage by respondents in spring 2020 (left; *n* = 111; question #11) and planned for summer and fall 2020 (right; *n* = 110; question #12)

**Figure 3:**
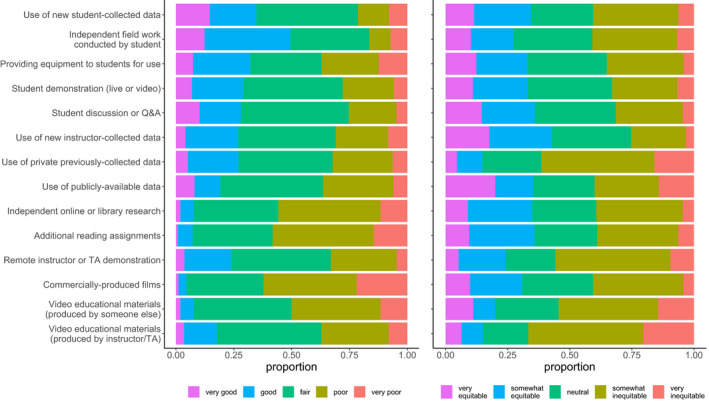
Respondent perceptions of the effectiveness of alternative remote substitute activities for field teaching (left; *n* = 116; question #13) and respondent perceptions of the equitability of alternative remote substitute activities for field teaching (right; *n* = 100; question #16)

**Figure 4:**
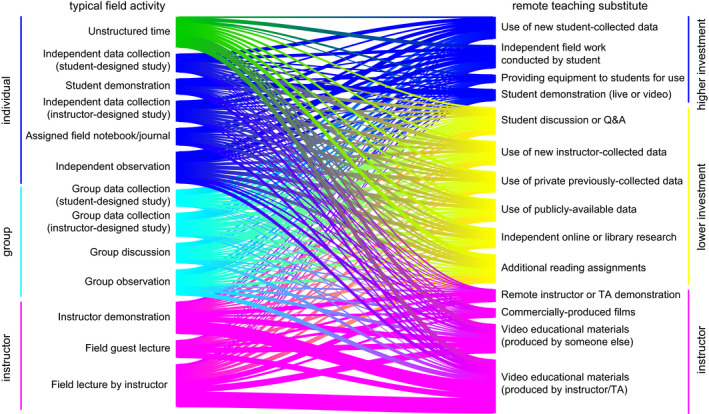
Sankey diagram mapping faculty actual or planned substitutions of typical field teaching activities (left) to remote teaching substitutes (right). Typical field activities are ordered from top to bottom by the author's subjective ranking of activities from most student‐centered to most instructor‐centered, and grouped in broad categories describing centeredness and student level of investment (*n* = 86; question #15)

Respondents reported typically teaching a diversity of field learning outcomes (Figure 1a) and a variety of field teaching techniques (Figure 1b), and extensive reductions or removal of field learning outcomes in response to modality shift (Figure 1a). Respondents reported using or planning to use a variety of remote teaching activities, on an ordinal 4‐category response item that ranged from “not at all” to “extensively” (Figure 2). Respondents reported variation in the effectiveness (Figure 3a) and equity (Figure 3b) of alternative remote substitute activities based on their responses to 5‐point Likert‐scale response items. Respondents used a tabular response item (see Supporting Information) to map face‐to‐face field activities to their substituted remote learning activities (Figure 4). I categorized, post hoc, free‐response answers provided to two questions that asked respondents to identify barriers to equity in teaching field topics face‐to‐face and in remote modalities (Table 1). Thirty‐seven respondents provided examples of what they considered successful remote adaptations of field teaching in response to a free‐response question, which I expanded and merged into three general approaches to remote teaching of field topics, organized by learning outcome type (Table 2).

**Table 1:** Most frequent post hoc categorizations of responses to the open‐ended questions “What are, in your opinion and experience, the largest barriers to inclusive teaching in typical field settings?”, at left (*n* = 99; question #17), and “What are, in your opinion and experience, the largest barriers to inclusive teaching when remote teaching field‐based topics?”, at right (*n* = 103; question #18)

Field Teaching Barrier	Frequency	Remote Teaching Barrier	Frequency
Experience	32	Technology	59
Accessibility	16	Student time	24
Student time	15	Less engaging modality	15
Social	12	Geography/ transportation	13
Equipment	13	Student–instructor connection	13
Comfort/ fear	10	Student experience or equipment	9
Transportation/ geography	10	Culture/race/ethnicity	6
Race/ethnicity	8	Instructor time	4
Expense	6	Safety	3
Class size	4	Community loss/ lack of groups	3
Instructor time	4	Lack of institutional support	2
There are no barriers	3	Accessibility	1

**Table 2:** Selected remote teaching activities reported by survey respondents and discretized into steps by the author, with learning outcomes, advantages, disadvantages, and resources (question #19)

Learning outcome type	Remote activity	Advantages	Disadvantages	Resource(s)
Identification and natural history	Assigned reading or video on identification Identification quiz with feedback (e.g. VL‐PI; Kirchoff, Delaney, Horton, & Dellinger‐Johnston, 2014) Students use iNaturalist to locate and attempt to identify organisms, or identify extant records Students receive feedback on identifications via peers and instructor Summative assessment	More active than simple photo quizzes, multiple opportunities for feedback	Requires student access to smartphone or camera, may require student ability to encounter and digitally record organisms of interest, may be applicable to limited range of organisms	Kirchoff et al. (2014), Unger, Rollins, Tietz, and Dumais (2020), iNaturalist
Field techniques	Instructor video demonstrating technique Follow‐up student question and answer session Student‐recorded video demonstrating technique Peers and instructor provide feedback on 3 Summative assessment	More active than simple demonstration, multiple opportunities for feedback, adaptable to wide variety of techniques	Requires student ability to record and upload video, availability of necessary materials and locations to students	Maloney, Storr, Morgan, and Ilic (2013)
Data collection and study design	Assess knowledge of field technique to be used Create student‐ or instructor‐designed sampling scheme and give or receive student feedback Students execute field data collection with instructor question and answer support Collate data using cloud‐based platform or participant science platform Use data in reflection, analysis lab(s), or summative assessment	Scaffolded exercise that builds off other knowledge, availability of alternative datasets or participant science data can guard against study failure	Somewhat complex implementation, availability of necessary materials and locations to students	Gastreich (2020)

## 4 DISCUSSION

Survey results revealed perceived negative effects of teaching modality shifts on field teaching during spring 2020 and upcoming semesters, which was unsurprising given that a large majority of respondents (79.5%) taught courses with modes of instruction impacted during spring 2020. The worsening public health situation in the United States during summer 2020 (Dong, Du, & Gardner, 2020) suggests that the smaller proportion of instructors expecting impacts to future semesters may have been optimistic. These negative effects included reduction or elimination of learning outcomes typically taught in field activities (Figure 1a), a shift to remote teaching activities that appear less student‐centered (Figures 2 and 4), and adoption of remote teaching activities that instructors viewed as relatively poor quality substitutes for field activities or that have substantial perceived shortcomings in terms of equity (Figure 3).

Respondents typically taught, in field settings, a variety of learning outcomes using diverse activities. The most frequently taught learning outcomes related to field techniques, data collection, natural history and identification, study design, and teamwork, and the most frequently used activities were instructor field lecture, group data collection in instructor‐designed studies, instructor demonstration, group observation, and independent observation (Figure 1). The learning outcomes most frequently reduced or eliminated in response to the pandemic were also those that were most commonly taught in field settings (Figure 1). Declining institutional support for field trips and increasing class enrollments likely had likely already largely forced field teaching to focus on learning outcomes difficult to teach by other means (Fleischner et al., 2017). This result further suggests that outcomes typically taught in field settings were difficult to replace in alternative modalities, especially given limited time for preparation and available information.

Respondents used or planned to use a diversity of remote teaching activities to substitute for activities typically taught in the field. The most frequently reported remote teaching activities used in spring 2020 were student discussion, video materials, additional reading assignments, independent research, or instructor demonstration (Figure 2). These activities appear to be both less student‐centered and less active than typical field activities, although this conclusion is contingent on the specific pedagogy applied (i.e. active learning can be incorporated into lectures or videos). Activities planned for use in future terms appeared to shift, to some extent, toward more active or student‐centered activities relative to those used in spring 2020 (Figure 3) which may result in improved student outcomes given the effectiveness of active learning (Freeman et al., 2014; Handelsman, Miller, & Pfund, 2007). Respondents mapped typical field activities to remote substitutes (Figure 4), and these results also suggested a shift from active, student‐centered activities to more instructor‐centered activities, although the survey did not directly ask respondents about active learning in remote teaching activities.

Respondents had generally negative views of both the effectiveness and equity of remote teaching activities (Figure 3). There was an apparent mismatch between perceived effectiveness (relatively high) and equity (relatively low) of independent data collection and fieldwork activities conducted by students. Free‐response answers to questions on barriers to equitable teaching suggested that while independent data collection and fieldwork activities were relatively effective substitutes for field teaching, they may be difficult to implement equitably in a remote modality. Identification of perceived barriers by respondents (Table 1) provides insight into what barriers to equity might be operating. Respondents also expressed relatively high perceived effectiveness and equity of instructor‐generated video lectures and demonstrations, which was surprising given the relative passivity of these types of exercises and the generally superior performance of more active pedagogical approaches (Freeman et al., 2014).

The sample of survey respondents from the complete US faculty population was nonrandom due to a combination of selection bias and likely response bias. One source of selection bias was my use of institutional websites to obtain email addresses for direct recruitment, because the numerous part‐time faculty and graduate students that teach a substantial portion of postsecondary courses may not be listed on such websites. This selection bias was probably only partially mitigated by distribution of the survey via email lists of professional societies. A commonly hypothesized source of response bias in faculty surveys on teaching is that faculty more engaged in their teaching responsibilities may be more likely to respond to surveys about their teaching (e.g., Becker & Watts, 2001). The substantial over‐representation of tenure‐track or tenured faculty in this survey (81% of respondents) is likely caused by these dual sources of bias. However, given the inductive nature of this survey, response bias may actually make the sample more useful in addressing the descriptive research questions, given that the respondents are likely to be tenure‐track or tenured faculty more engaged in their teaching. Thus, these data may represent more useful and thoughtful responses than selected nonrespondents might have provided.

Teaching field learning outcomes in a remote modality clearly poses pedagogical and logistical challenges. Respondents offered a variety of potentially successful approaches to remote teaching of topics typically taught in the field, several of which I summarized, expanded, and related to selected literature (Table 2). I focused on the learning outcome types most frequently taught in typical field settings and most heavily impacted by modality shift: identification and natural history, field techniques, data collection, and study design. The suggested activities and related resources are general rather than specific and may be applicable to a variety of synchronous or asynchronous remote courses that teach such learning outcomes. I assumed that more active and student‐centered activities are generally more engaging to students and likely to produce positive outcomes in both face‐to‐face (Freeman et al., 2014) and remote environments (Farrel et al., 2018). I do not discuss virtual field trips as a substitute for field activities, because virtual field trips do not appear to represent a single pedagogical approach, but rather a wide variety of remote or even face‐to‐face activities that are meant to substitute for the traditional field trip.

The challenges to inclusive teaching posed by shifting to distance‐learning modalities that were most frequently identified by respondents were technology, student time, less engaging modality, and geography or transportation (Table 1). A combination of institutional support, such as providing necessary equipment to students, and thoughtful remote course design, such as focusing on activities likely to be effective in a remote environment, may assist students in overcoming these faculty‐perceived barriers. An important consideration, expressed unprompted by 27 survey respondents in free‐response questions, is that remote teaching modalities may exacerbate existing inequalities between students, presumably because of correlation between access to technology and socioeconomic class or other factors (Table 1). Further, asking students to engage in field activities alone may present personal hazards to students, and risk could be correlated with socioeconomic class, ability, or any number of other factors. Mitigation of these hazards is worth considering when designing inclusive courses.

The faculty survey results and discussion presented here represent a first attempt at applying survey‐based approaches to understanding and improving field pedagogy within a sudden, seemingly intractable disruption that has uniquely impacted field‐based higher education in ecology and evolution. This survey was designed, administered, and analyzed in relatively short order, leading to several potential shortcomings that can be overcome through more targeted and well‐designed education research. Future studies with improved randomization during selection and elimination of response bias would improve inferential scale and confidence. More targeted research that specifically assesses the application and effectiveness of active learning strategies in remote or face‐to‐face teaching of field learning outcomes would allow for more specific pedagogical recommendations. I optimistically hope that the self‐reflection and assessment of existing field teaching activities forced by the pandemic will spur additional research into field pedagogy in ecology and evolution, and in the long run, improved and more inclusive experiences for students in field‐based disciplines.

## CONFLICT OF INTEREST

The author has no conflicts of interest to declare.

## AUTHOR CONTRIBUTION

**Daniel Barton:** Conceptualization (lead); Data curation (lead); Formal analysis (lead); Investigation (lead); Methodology (lead); Project administration (lead); Resources (lead); Software (lead); Supervision (lead); Validation (lead); Visualization (lead); Writing‐original draft (lead); Writing‐review & editing (lead).

## Supporting information

Supinfo

## Data Availability

Summarized and aggregated survey data that support the findings of this study are available in the Supporting Information of this article. All individually identifying information has been removed, and long‐form answers containing individually identified information have not been shared because informed consent to do so was not obtained from respondents.
