# Supplementary material for: Impacts of the COVID‐19 pandemic on field instruction and remote teaching alternatives: Results from a survey of instructors
Source: Ecol Evol. 2020 Aug 7;10(22):12499–507. doi: 10.1002/ece3.6628 (PMC7436523; doi:10.1002/ece3.6628)
Supplement: Supplementary file 1 — Supinfo [file ECE3-10-12499-s001.pdf]

Supporting Information for "Impacts of the COVID-19 pandemic on field instruction and remote-teaching alternatives: results from a survey of instructors", Daniel C. Barton.

Contact emails, survey questions, and aggregated and anonymized survey data.

The following contact email was sent to email listservs during April 2020. Participants recruited via direct contact received an email from the SurveyMonkey platform containing the informed consent statement.

"My name is Daniel Barton and I am a faculty member at Humboldt State University. I write to invite you to participate in my research study on how college instructors in the United States are teaching field-based topics remotely during the COVID-19 pandemic.

If you decide to participate in this study, you will be asked to complete an online survey that will take approximately 20 minutes to complete. Participation is completely voluntary, and your answers will be confidential. Please feel free to share this email and the link below with colleagues that you believe would be interested in completing this survey.

Remember, this is completely voluntary, and you can choose to be in the study or not. If you'd like to participate, please use this link: https://www.surveymonkey.com/r/field-and-remote-teaching-during-covid-pandemic

Thank you very much.

Sincerely,
Dan Barton

--
Daniel C. Barton, PhD
Associate Professor, Quantitative Population Ecology
Department of Wildlife
Humboldt State University
1 Harpst Street
Arcata, California 95521
daniel.barton@humboldt.edu
(707) 826-3430"

Field and remote teaching during the COVID-19 pandemic

Informed consent

* 1. You are invited to participate in a research study which will involve responding to an online survey. My name is Daniel Barton, and I am an Associate Professor at Humboldt State University. The purpose of this research is to survey how college-level instructors are responding to changes in modes of instruction forced by the COVID-19 pandemic when teaching topics typically taught in field settings. If you decide to participate, you will be asked to answer 25 survey questions. Your participation in this study will last approximately 20 minutes. There are no anticipated risks involved for participants. There are some potential benefits to this research, particularly providing timely information on how educators that teach in field settings are confronting the unique set of challenges imposed by a global pandemic. Your participation in this project is voluntary. You have the right not to participate at all or to leave the study at any time without penalty or loss of benefits to which you may otherwise be entitled. If you choose to withdrawal while completing the survey, simply do not submit your responses and close your browser window.

Any information that is obtained in connection with this study and that can be identified with you will remain confidential and will be disclosed only with your permission. Measures to ensure your confidentiality will include providing only a single person access to individually-identifiable information, and removing the association between individual responses and participant names immediately after data collection. The individually-identifiable data obtained will be maintained in a safe location, separate from survey responses, and will be destroyed after a period of 3 years after the study is completed. This consent form will be maintained in a safe location and will be destroyed after a period of 3 years after the study is completed. If you have any questions about this research at any time, please email me at daniel.barton@humboldt.edu. If you have any concerns with this study or questions about your rights as a participant, contact the Institutional Review Board for the Protection of Human Subjects at irb@humboldt.edu or (707) 826-5165. Please print this informed consent form now and retain it for your future reference. If you agree to voluntarily participate in this research as described, (and are at least 18 years old), please indicate that below to begin the survey. Thank you for your participation in this research.

- ○ I have read and understood this consent information, and agree to participate in this study.
- ○ I do not want to participate in this study.

Field and remote teaching during the COVID-19 pandemic

Survey

2. Please select the option(s) that best apply to you:

- [ ] Currently (Spring 2020) instructor of a US college-level course or course module (such as a lab) with field components for which the mode of instruction has been impacted by the COVID-19 pandemic
- [ ] Plan to (Summer/Fall 2020) be instructor of a US college-level course or course module (such as a lab) with field components for which you anticipate the mode of instruction will be impacted by the COVID-19 pandemic
- [ ] Currently (Spring 2020) the instructor of a US college-level course with field components that was **already** being taught largely or wholly taught remotely before the impacts of the COVID-19 pandemic
- [ ] Plan to (in Summer/Fall 2020) be instructor of a US college-level course or course module (such as a lab) with field components that was **already** planned as largely or wholly taught remotely before the impacts of the COVID-19 pandemic
- [ ] None of the above options apply, but you instruct, have instructed, or develop instructional materials for a US college-level course with field components, that are taught either in-person or remotely.

3. What discipline do you **primarily** teach in field-based settings?

4. The **assessed** learning outcomes or objectives of your course(s) that include field components as typically taught are best described as:

- ○ Not dependent on field components
- ○ Minimally dependent on field components
- ○ Largely dependent on field components
- ○ Wholly dependent on field components

5. The course(s) with field components you teach are mostly composed of (select all that apply):

- [ ] First-year students
- [ ] Second-year students
- [ ] Third-year students
- [ ] Fourth-year students (or beyond)
- [ ] Graduate students

6. The field components in your course(s) before any COVID-19 impacts included (select all that apply):

- [ ] Short field trips
- [ ] Day-long field trips
- [ ] Short (<3 night) overnight field trips
- [ ] Long (> 3 night) overnight field trips
- [ ] The course is largely or entirely taught in the field or at a field station
- [ ] Supervised field work conducted by students during field trips
- [ ] Independent field work conducted by students on their own time

7. Which of the following best describe learning outcomes or objectives met partly or wholly through field instruction in your course(s) **as typically taught** and that are assessed (i.e. tested or otherwise measured) when evaluating students? Select all applicable answers.

- [ ] Natural history and identification
- [ ] Spatial data collection or application
- [ ] Safety, orienteering, or navigation
- [ ] Field techniques or methods
- [ ] Teamwork or group dynamics
- [ ] Study or sampling design
- [ ] Data collection, keeping a field journal, or note-taking
- [ ] Deductive reasoning or hypothesis testing
- [ ] Inductive reasoning and/or retroduction
- [ ] Other (please specify)

8. Which methods of field instruction did you typically (i.e. pre-COVID-19) use for **assessed** learning outcomes?

- [ ] Unstructured time
- [ ] Field lecture by instructor
- [ ] Field guest lecture
- [ ] Group observation
- [ ] Independent observation
- [ ] Instructor demonstration
- [ ] Student demonstration
- [ ] Group discussion
- [ ] Assigned field notebook/journal
- [ ] Group data collection (instructor-designed study)
- [ ] Independent data collection (instructor-designed study)
- [ ] Group data collection (student-designed study)
- [ ] Independent data collection (student-designed study)
- [ ] Other (please specify)

9. In response to the COVID-19 pandemic, have you or do you plan to (select all that apply):

- [ ] Remove field-based learning outcomes or objectives of my course to accommodate a changed mode of instruction
- [ ] Reduce field-based learning outcomes or objectives of my course to accommodate a changed mode of instruction
- [ ] Switch from teaching field-based learning outcomes in the field to teaching them remotely
- [ ] Switch from teaching field-based learning outcomes in person in the field to teaching them remotely - but still in the field
- [ ] Made no changes, because I was already teaching remotely

10. Which of the following best describes learning outcomes or objectives typically met partly or wholly through field instruction in your course(s) **that have been removed or reduced** (or that you plan to remove or reduce) in response to the COVID-19 pandemic:

- [ ] Natural history and identification
- [ ] Spatial data collection or application
- [ ] Safety, orienteering, or navigation
- [ ] Field techniques or methods
- [ ] Teamwork or group dynamics
- [ ] Study or sampling design
- [ ] Data collection, keeping a field journal, or note-taking
- [ ] Deductive reasoning or hypothesis testing
- [ ] Inductive reasoning and/or retroduction
- [ ] I have not removed or reduced learning outcomes in response to COVID-19
- [ ] Other (please specify)

11. How much have you **already used** the following approaches or materials to replace approaches or materials that you would typically conduct in the field?

	Not at all	A little	A moderate amount	Extensively
Video educational materials (produced by instructor/TA)	○	○	○	○
Video educational materials (produced by someone else)	○	○	○	○
Commercially-produced films	○	○	○	○
Additional reading assignments	○	○	○	○
Independent online or library research	○	○	○	○
Use of publicly-available data	○	○	○	○
Use of private previously-collected data	○	○	○	○
Use of new instructor-collected data	○	○	○	○
Use of new student-collected data	○	○	○	○
Remote instructor or TA demonstration	○	○	○	○
Independent field work conducted by student	○	○	○	○
Providing equipment to students	○	○	○	○
Student demonstration (live or video)	○	○	○	○
Student discussion or Q&A	○	○	○	○

12. How much do you **plan to use** the following approaches or materials to replace approaches or materials that you would typically conduct in the field?

	Not at all	A little	A moderate amount	Extensively
Video educational materials (produced by instructor/TA)	○	○	○	○
Video educational materials (produced by someone else)	○	○	○	○
Commercially-produced films	○	○	○	○
Additional reading assignments	○	○	○	○
Independent online or library research	○	○	○	○
Use of publicly-available data	○	○	○	○
Use of private previously-collected data	○	○	○	○
Use of new instructor-collected data	○	○	○	○
Use of new student-collected data	○	○	○	○
Remote instructor or TA demonstration	○	○	○	○
Independent field work conducted by student	○	○	○	○
Providing equipment to students	○	○	○	○
Student demonstration (live or video)	○	○	○	○
Student discussion or Q&A	○	○	○	○

13. In your experience, how effective do you think the following approaches or materials are as substitutes for teaching you would typically conduct in the field?

	Very Poor Substitute	Poor Substitute	Fair Substitute	Good Substitute	Very Good Substitute	NA / No experience
Video educational materials (produced by instructor/TA)	◯	◯	◯	◯	◯	◯
Video educational materials (produced by someone else)	◯	◯	◯	◯	◯	◯
Commercially-produced films	◯	◯	◯	◯	◯	◯
Additional reading assignments	◯	◯	◯	◯	◯	◯
Independent online or library research	◯	◯	◯	◯	◯	◯
Use of publicly-available data	◯	◯	◯	◯	◯	◯
Use of private previously-collected data	◯	◯	◯	◯	◯	◯
Use of new instructor-collected data	◯	◯	◯	◯	◯	◯
Use of new student-collected data	◯	◯	◯	◯	◯	◯
Remote instructor or TA demonstration	◯	◯	◯	◯	◯	◯
Independent field work conducted by student	◯	◯	◯	◯	◯	◯
Providing equipment to students	◯	◯	◯	◯	◯	◯
Student demonstration (live or video)	◯	◯	◯	◯	◯	◯
Student discussion or Q&A	◯	◯	◯	◯	◯	◯

14. In the context of typical field experiences, how equitable do you think the following teaching methods or materials are across students with diverse backgrounds (including but not limited to race, ethnicity, gender, economic class, and age)?

	Very inequitable	Somewhat inequitable	Neutral	Somewhat equitable	Very equitable
Unstructured time	◯	◯	◯	◯	◯
Field lecture by instructor	◯	◯	◯	◯	◯
Field guest lecture	◯	◯	◯	◯	◯
Group observation	◯	◯	◯	◯	◯
Independent observation	◯	◯	◯	◯	◯
Instructor demonstration	◯	◯	◯	◯	◯
Student demonstration	◯	◯	◯	◯	◯
Group discussion	◯	◯	◯	◯	◯
Assigned field notebook/journal	◯	◯	◯	◯	◯
Group data collection (instructor-designed study)	◯	◯	◯	◯	◯
Independent data collection (instructor-designed study)	◯	◯	◯	◯	◯
Group data collection (student-designed study)	◯	◯	◯	◯	◯
Independent data collection (student-designed study)	◯	◯	◯	◯	◯

15. If you have substituted remote (online) teaching for field teaching, how have you substituted? Match field teaching methods (in **rows**) with potential remote (online) substitutes (in **columns**) by selecting any checkboxes that apply.

	Video educational materials (produced by instructor/TA)	Video educational materials (produced by someone else)	Commercially-produced films	Additional reading assignments	Independent online or library research	Use of publicly-available data	Use of private previously-collected data	Use of new instructor-collected data	Use of new student-collected data	Remote instructor or TA demonstration	Independent field work conducted by student	Providing equipment to students for use	Stude demonstr (live or v
Unstructured time	☐	☐	☐	☐	☐	☐	☐	☐	☐	☐	☐	☐	☐
Field lecture by instructor	☐	☐	☐	☐	☐	☐	☐	☐	☐	☐	☐	☐	☐
Field guest lecture	☐	☐	☐	☐	☐	☐	☐	☐	☐	☐	☐	☐	☐
Group observation	☐	☐	☐	☐	☐	☐	☐	☐	☐	☐	☐	☐	☐
Independent observation	☐	☐	☐	☐	☐	☐	☐	☐	☐	☐	☐	☐	☐
Instructor demonstration	☐	☐	☐	☐	☐	☐	☐	☐	☐	☐	☐	☐	☐
Student demonstration	☐	☐	☐	☐	☐	☐	☐	☐	☐	☐	☐	☐	☐
Group discussion	☐	☐	☐	☐	☐	☐	☐	☐	☐	☐	☐	☐	☐
Assigned field notebook/journal	☐	☐	☐	☐	☐	☐	☐	☐	☐	☐	☐	☐	☐
Group data collection (instructor-designed study)	☐	☐	☐	☐	☐	☐	☐	☐	☐	☐	☐	☐	☐
Independent data collection (instructor-designed study)	☐	☐	☐	☐	☐	☐	☐	☐	☐	☐	☐	☐	☐
Group data collection (student-designed study)	☐	☐	☐	☐	☐	☐	☐	☐	☐	☐	☐	☐	☐
Independent data collection (student-designed study)	☐	☐	☐	☐	☐	☐	☐	☐	☐	☐	☐	☐	☐

Other substitutions not shown (please specify)

16. In the context of substituting typical field experiences with remote teaching, how equitable do you think the following remote teaching methods or materials are across students with diverse backgrounds (including but not limited to race, ethnicity, gender, economic class, and age)?

	Very inequitable	Somewhat inequitable	Neutral	Somewhat equitable	Very equitable	N/A
Video educational materials (produced by instructor/TA)	○	○	○	○	○	○
Video educational materials (produced by someone else)	○	○	○	○	○	○
Commercially-produced films	○	○	○	○	○	○
Additional reading assignments	○	○	○	○	○	○
Independent online or library research	○	○	○	○	○	○
Use of publicly-available data	○	○	○	○	○	○
Use of private previously-collected data	○	○	○	○	○	○
Use of new instructor-collected data	○	○	○	○	○	○
Use of new student-collected data	○	○	○	○	○	○
Remote instructor or ta demonstration	○	○	○	○	○	○
Independent field work conducted by student	○	○	○	○	○	○
Providing equipment to students	○	○	○	○	○	○
Student demonstration (live or video)	○	○	○	○	○	○
Student discussion or Q&A	○	○	○	○	○	○

17. What are, in your opinion and experience, the largest barriers to inclusive teaching in typical field settings?

18. What are, in your opinion and experience, the largest barriers to inclusive teaching when remote teaching field-based topics?

19. Please briefly describe a successful, or planned, adaptation of field teaching to a remote (online) teaching environment.

20. Please briefly describe what you see as the largest challenge to adapting field teaching to a remote (online) teaching environment.

21. Which Carnegie Classification best describes your primary institution?

- Doctoral university
- Master's college or university
- Baccalaureate college
- Baccalaureate/associate's college
- Associate's college
- Special focus: two-year
- Special focus: four-year
- Tribal college

22. Which best describes your primary institution?

- Public
- Private

23. Which best describes your primary position at your primary institution?

- Graduate student
- Lecturer or adjunct
- Post-doctoral associate
- Research faculty
- Tenure-track or tenured faculty
- Instructional support or faculty development
- Administrator

24. Name (optional):

First

Last

25. Email address (optional):

26. I would like to be contacted with a follow-up report of aggregated data from this survey:

- Yes
- No

Aggregated and anonymized survey data

Q1. (informed consent)

Response P	Responses	Answer Choices
100.0%	117	I have read and understood this consent information, and agree to participate in this study.
0.0%	0	I do not want to participate in this study.
Answered	117	
Skipped	0	

Q2. Please select the option(s) that best apply to you:

Response P	Responses	Answer Choices
79.49%	93	Currently (Spring 2020) instructor of a US college-level course or course module (such as a lab) with field components for which the mode of instruction has been impacted by the COVID-19 pandemic
45.3%	53	Plan to (Summer/Fall 2020) be instructor of a US college-level course or course module (such as a lab) with field components for which you anticipate the mode of instruction will be impacted by the COVID-19 pandemic
2.56%	3	Currently (Spring 2020) the instructor of a US college-level course with field components that was already being taught largely or wholly taught remotely before the impacts of the COVID-19 pandemic
4.27%	5	Plan to (in Summer/Fall 2020) be instructor of a US college-level course or course module (such as a lab) with field components that was already planned as largely or wholly taught remotely before the impacts of the COVID-19 pandemic
5.13%	6	None of the above options apply, but you instruct, have instructed, or develop instructional materials for a US college-level course with field components, that are taught either in-person or remotely.
Answered	117	
Skipped	0	

Q3. What discipline do you primarily teach in field-based settings?

Response P	Responses	Answer Choices
11.21%	12	Geology or related disciplines
1.87%	2	Hydrology or related disciplines
0.0%	0	Meterology or related disciplines
0.0%	0	Paleontology or related disciplines
18.69%	20	General ecology or related disciplines
24.3%	26	Wildlife biology or related disciplines
3.74%	4	Vertebrate Zoology or related disciplines
1.87%	2	Invertebrate Zoology or related disciplines
0.0%	0	Disease ecology or related disciplines
3.74%	4	Fisheries or related disciplines
1.87%	2	Oceanography or related disciplines
5.61%	6	Botany or related disciplines
11.21%	12	Forestry or related disciplines
0.0%	0	Range science or related disciplines
1.87%	2	Soil science or related disciplines
0.93%	1	Cellular or microbiology or related disciplines
0.0%	0	Health sciences
2.8%	3	Outdoor education, recreation, leadership, or related disciplines
10.28%	11	Other (please specify)
Answered	107	
Skipped	10	

Q4. The assessed learning outcomes or objectives of your course(s) that include field components as typically taught are best described as:

Response P	Responses	Answer Choices
0.86%	1	Not dependent on field components
27.59%	32	Minimally dependent on field components
63.79%	74	Largely dependent on field components
7.76%	9	Wholly dependent on field components
Answered	116	
Skipped	1	

Q5. The course(s) with field components you teach are mostly composed of (select all that apply):

Response P	Responses	Answer Choices
18.1%	21	First-year students
37.07%	43	Second-year students
51.72%	60	Third-year students
62.93%	73	Fourth-year students (or beyond)
18.1%	21	Graduate students
Answered	116	
Skipped	1	

Q6. The field components in your course(s) before any COVID-19 impacts included (select all that apply):

Response P	Responses	Answer Choices
77.59%	90	Short field trips
36.21%	42	Day-long field trips
23.28%	27	Short (<3 night) overnight field trips
12.93%	15	Long (>3 night) overnight field trips
20.69%	24	The course is largely or entirely taught in the field or at a field station
41.38%	48	Supervised field work conducted by students during field trips
38.79%	45	Independent field work conducted by students on their own time
Answered	116	
Skipped	1	

Q7. Which of the following best describe learning outcomes or objectives met partly or wholly through field instruction in your course(s) as typically taught and that are assessed (i.e. tested or otherwise measured) when evaluating students? Select all applicable answers.

Response P	Responses	Answer Choices
67.24%	78	Natural history and identification
40.52%	47	Spatial data collection or application
24.14%	28	Safety, orienteering, or navigation
85.34%	99	Field techniques or methods
55.17%	64	Teamwork or group dynamics
56.03%	65	Study or sampling design
77.59%	90	Data collection, keeping a field journal, or note-taking
45.69%	53	Deductive reasoning or hypothesis testing
19.83%	23	Inductive reasoning and/or retroduction
9.48%	11	Other (please specify)
Answered	116	
Skipped	1	

Q8. Which methods of field instruction did you typically (i.e. pre-COVID-19) use for assessed learning outcomes?

Response P	Responses	Answer Choices
19.3%	22	Unstructured time
65.79%	75	Field lecture by instructor
35.96%	41	Field guest lecture
58.77%	67	Group observation
50.0%	57	Independent observation
59.65%	68	Instructor demonstration
23.68%	27	Student demonstration
42.11%	48	Group discussion
48.25%	55	Assigned field notebook/journal
61.4%	70	Group data collection (instructor-designed study)
33.33%	38	Independent data collection (instructor-designed study)
25.44%	29	Group data collection (student-designed study)

28.95%	33	Independent data collection (student-designed study)
4.39%	5	Other (please specify)
Answered	114	
Skipped	3	

Q9. In response to the COVID-19 pandemic, have you or do you plan to (select all that apply):

Response P	Responses	Answer Choices
28.95%	33	Remove field-based learning outcomes or objectives of my course to accommodate a changed mode of instruction
46.49%	53	Reduce field-based learning outcomes or objectives of my course to accommodate a changed mode of instruction
57.02%	65	Switch from teaching field-based learning outcomes in the field to teaching them remotely
32.46%	37	Switch from teaching field-based learning outcomes in person in the field to teaching them remotely - but still in the field
0.88%	1	Made no changes, because I was already teaching remotely
Answered	114	
Skipped	3	

Q10. Which of the following best describes learning outcomes or objectives typically met partly or wholly through field instruction in your course(s) that have been removed or reduced (or that you plan to remove or reduce) in response to the COVID-19 pandemic:

Response P	Responses	Answer Choices
41.96%	47	Natural history and identification
23.21%	26	Spatial data collection or application
19.64%	22	Safety, orienteering, or navigation
65.18%	73	Field techniques or methods
41.07%	46	Teamwork or group dynamics
27.68%	31	Study or sampling design
50.89%	57	Data collection, keeping a field journal, or note-taking
18.75%	21	Deductive reasoning or hypothesis testing
7.14%	8	Inductive reasoning and/or retroduction
8.93%	10	I have not removed or reduced learning outcomes in response to COVID-19
8.93%	10	Other (please specify)
Answered	112	
Skipped	5	

Q11. How much have you already used the following approaches or materials to replace approaches or materials that you would typically conduct in the field?

Not at all	A little	A moderate	Extensively	Total	Weighted A	Answer Choices
33	15	30	28	106	2.5	Video educational materials (produced by instructor/TA)
34	36	25	10	105	2.1	Video educational materials (produced by someone else)
75	23	6	0	104	1.34	Commercially-produced films
36	32	28	9	105	2.1	Additional reading assignments
39	28	28	5	100	1.99	Independent online or library research
45	29	19	9	102	1.92	Use of publicly-available data
47	23	16	13	99	1.95	Use of private previously-collected data
61	16	11	14	102	1.78	Use of new instructor-collected data
60	21	11	7	99	1.65	Use of new student-collected data
48	24	17	14	103	1.97	Remote instructor or TA demonstration
54	20	20	8	102	1.82	Independent field work conducted by student
71	12	8	9	100	1.55	Providing equipment to students
75	12	7	4	98	1.39	Student demonstration (live or video)
24	28	35	18	105	2.45	Student discussion or Q&A
				Answered	111	
				Skipped	6	

Q12. How much do you plan to use the following approaches or materials to replace approaches or materials that you would typically conduct in the field?

Not at all	A little	A moderate	Extensively	Total	Weighted A	Answer Choices
19	18	38	33	108	2.79	Video educational materials (produced by instructor/TA)
32	30	27	15	104	2.24	Video educational materials (produced by someone else)
59	29	12	1	101	1.55	Commercially-produced films
30	32	31	11	104	2.22	Additional reading assignments
32	36	32	3	103	2.06	Independent online or library research
39	22	30	14	105	2.18	Use of publicly-available data
51	20	22	10	103	1.91	Use of private previously-collected data
57	20	13	11	101	1.78	Use of new instructor-collected data
56	29	8	8	101	1.68	Use of new student-collected data
36	24	30	14	104	2.21	Remote instructor or TA demonstration
49	22	20	12	103	1.95	Independent field work conducted by student
72	15	8	3	98	1.41	Providing equipment to students
54	31	12	3	100	1.64	Student demonstration (live or video)
17	26	34	27	104	2.68	Student discussion or Q&A
				Answered	110	
				Skipped	7	

Q13. In your experience, how effective do you think the following approaches or materials are as substitutes for teaching you would typically conduct in the field?

Very Poor S	Poor Substi	Fair Substit	Good Subst	Very Good	NA / No ex	Total	Weighted A	Answer Choices
9	33	51	16	4	3	116	2.82	Video educational materials (produced by instructor/TA)
12	40	44	6	2	8	112	2.66	Video educational materials (produced by someone else)
19	35	29	3	1	23	110	2.8	Commercially-produced films
16	48	38	7	1	3	113	2.42	Additional reading assignments
12	46	38	6	2	9	113	2.63	Independent online or library research
6	30	44	11	8	14	113	3.12	Use of publicly-available data
6	25	39	21	5	15	111	3.22	Use of private previously-collected data
8	22	41	22	4	14	111	3.18	Use of new instructor-collected data
7	12	39	18	13	21	110	3.55	Use of new student-collected data
5	31	47	22	4	3	112	2.96	Remote instructor or TA demonstration
7	9	33	36	12	15	112	3.6	Independent field work conducted by student
10	20	25	20	6	30	111	3.47	Providing equipment to students
5	19	37	19	6	26	112	3.48	Student demonstration (live or video)
5	22	50	19	11	5	112	3.17	Student discussion or Q&A
						Answered	116	
						Skipped	1	

Q14. In the context of typical field experiences, how equitable do you think the following teaching methods or materials are across students with diverse backgrounds (including but not limited to race, ethnicity, gender, economic class, and age)?

Very inequi	Somewhat i	Neutral	Somewhat e	Very equita	Total	Answer Choices
10	41	36	14	8	109	Unstructured time
0	19	27	37	27	110	Field lecture by instructor
1	13	39	37	19	109	Field guest lecture
3	23	31	40	13	110	Group observation
3	31	35	25	14	108	Independent observation
0	10	32	43	26	111	Instructor demonstration
5	28	38	27	10	108	Student demonstration
3	34	38	25	9	109	Group discussion
1	16	30	48	16	111	Assigned field notebook/journal
2	30	31	35	12	110	Group data collection (instructor-designed study)
5	32	31	30	12	110	Independent data collection (instructor-designed study)
9	32	34	26	9	110	Group data collection (student-designed study)
9	37	35	19	10	110	Independent data collection (student-designed study)
				Answered	111	

Skipped	6

Q15. If you have substituted remote (online) teaching for field teaching, how have you substituted? Match field teaching methods (in rows) with potential remote (online) substitutes (in columns) by selecting any checkboxes that apply.

Video educa	Video educa	Commercia	Additional r	Independen	Use of publ	Use of priva	Use of new	Use of new	Remote inst	Independen	Providing e	Student den	Student disc	Total	Answer Choices
18	15	6	14	8	4	6	5	3	4	10	4	1	4	44	Unstructured time
49	20	5	13	6	10	4	7	2	16	2	0	0	8	68	Field lecture by instructor
16	16	6	7	0	3	0	1	0	5	0	0	0	4	40	Field guest lecture
13	9	5	7	4	6	8	3	5	3	7	3	4	11	47	Group observation
10	7	3	8	11	10	11	7	14	0	14	5	2	5	54	Independent observation
42	17	4	6	2	5	2	8	0	12	2	1	1	6	61	Instructor demonstration
8	4	1	4	0	0	2	1	3	0	2	3	16	8	29	Student demonstration
8	2	0	7	4	5	4	3	4	2	2	0	4	28	46	Group discussion
8	2	2	10	5	4	4	5	6	0	8	1	0	3	33	Assigned field notebook/journal
3	2	0	6	7	8	15	11	10	0	8	3	3	4	38	Group data collection (instructor-designed study)
2	2	0	5	7	7	8	9	9	0	11	4	2	4	36	Independent data collection (instructor-designed study)
0	1	0	4	7	7	7	10	7	0	6	1	3	3	27	Group data collection (student-designed study)
1	1	0	4	8	7	4	11	10	0	8	2	3	3	32	Independent data collection (student-designed study)
														16	Other substitutions not shown (please specify)
													Answered	86	
													Skipped	31	

Q16. In the context of substituting typical field experiences with remote teaching, how equitable do you think the following remote teaching methods or materials are across students with diverse backgrounds (including but not limited to race, ethnicity, gender, economic class, and age)?

Very inequi	Somewhat i	Neutral	Somewhat e	Very equita	N/A	Total	Weighted A	Answer Choices
6	31	24	25	9	5	100	3	Video educational materials (produced by instructor/TA)
4	33	26	19	9	8	99	2.96	Video educational materials (produced by someone else)
6	30	28	15	9	11	99	2.9	Commercially-produced films
4	30	31	20	12	3	100	3.06	Additional reading assignments
9	44	19	18	5	5	100	2.64	Independent online or library research
6	24	31	20	10	9	100	3.04	Use of publicly-available data
4	24	29	19	13	11	100	3.15	Use of private previously-collected data
3	20	29	23	16	8	99	3.32	Use of new instructor-collected data
13	36	23	8	10	9	99	2.62	Use of new student-collected data
4	31	23	23	8	7	96	3	Remote instructor or ta demonstration
19	43	17	8	6	6	99	2.34	Independent field work conducted by student
12	22	21	13	17	12	97	3.01	Providing equipment to students
14	40	21	9	4	11	99	2.42	Student demonstration (live or video)
6	33	24	22	11	4	100	2.99	Student discussion or Q&A
						Answered	100	
						Skipped	17	

Q17. What are, in your opinion and experience, the largest barriers to inclusive teaching in typical field settings?

99	Answered
18	Skipped

Q18. What are, in your opinion and experience, the largest barriers to inclusive teaching when remote teaching field-based topics?

103	Answered
14	Skipped

Q19. Please briefly describe a successful, or planned, adaptation of field teaching to a remote (online) teaching environment.

86	Answered

31	Skipped

Q20. Please briefly describe what you see as the largest challenge to adapting field teaching to a remote (online) teaching environment.

96	Answered
21	Skipped

Q21. Which Carnegie Classification best describes your primary institution?

Response P	Responses	Answer Choices
44.35%	51	Doctoral university
25.22%	29	Master's college or university
20.87%	24	Baccalaureate college
2.61%	3	Baccalaureate/associate's college
6.96%	8	Associate's college
0.0%	0	Special focus: two-year
0.0%	0	Special focus: four-year
0.0%	0	Tribal college
Answered	115	
Skipped	2	

Q22. Which best describes your primary institution?

Response P	Responses	Answer Choices
80.0%	92	Public
20.0%	23	Private
Answered	115	
Skipped	2	

Q23. Which best describes your primary position at your primary institution?

Response P	Responses	Answer Choices
3.48%	4	Graduate student
9.57%	11	Lecturer or adjunct
0.87%	1	Post-doctoral associate
3.48%	4	Research faculty
80.87%	93	Tenure-track or tenured faculty
0.87%	1	Instructional support or faculty development
0.87%	1	Administrator
Answered	115	
Skipped	2	

Q24. Name (optional):

Response P	Responses	Answer Choices
100.0%	70	First
100.0%	70	Last
Answered	70	
Skipped	47	

Q25. Email address (optional):

74	Answered
43	Skipped

Q26. I would like to be contacted with a follow-up report of aggregated data from this survey:

Response P	Responses	Answer Choices
72.55%	74	Yes
27.45%	28	No
Answered	102	
Skipped	15	
